# Dietary supplementation with ketoacids protects against CKD-induced oxidative damage and mitochondrial dysfunction in skeletal muscle of 5/6 nephrectomised rats

**DOI:** 10.1186/s13395-018-0164-z

**Published:** 2018-05-31

**Authors:** Dongtao Wang, Lianbo Wei, Yajun Yang, Huan Liu

**Affiliations:** 1 Department of Traditional Chinese Medicine, Shenzhen Hospital, Southern Medical University, Shenzhen, 518000 Guangdong China; 2 Department of Nephrology, Shenzhen Traditional Chinese Medicine Hospital, Guangzhou University of Traditional Chinese Medicine, Shenzhen, 518033 Guangdong China; 3 Department of Nephrology, Ruikang Affiliated Hospital, Guangxi University of Chinese Medicine, Nanning, 530011 Guangxi China; 4 Department of Pharmacology, Guangdong Key Laboratory for R&D of Natural Drug, Guangdong Medical University, Zhanjiang, 524023 Guangdong China

**Keywords:** Chronic kidney disease, Muscle atrophy, Ketoacids, Oxidative stress, Mitochondrial dysfunction

## Abstract

**Background:**

A low-protein diet supplemented with ketoacids (LPD + KA) maintains the nutritional status of patients with chronic kidney disease (CKD). Oxidative damage and mitochondrial dysfunction associated with the upregulation of p66SHC and FoxO3a have been shown to contribute to muscle atrophy. This study aimed to determine whether LPD + KA improves muscle atrophy and attenuates the oxidative stress and mitochondrial damage observed in CKD rats.

**Methods:**

5/6 nephrectomy rats were randomly divided into three groups and fed with either 22% protein (normal-protein diet; NPD), 6% protein (low-protein diets; LPD) or 5% protein plus 1% ketoacids (LPD + KA) for 24 weeks. Sham-operated rats with NPD intake were used as the control.

**Results:**

KA supplementation improved muscle atrophy and function in CKD + LPD rats. It also reduced the upregulation of genes related to the ubiquitin-proteasome system and 26S proteasome activity, as well as protein and mitochondrial oxidative damage in the muscles of CKD + LPD rats. Moreover, KA supplementation prevented the drastic decrease in activities of mitochondrial electron transport chain complexes, mitochondrial respiration, and content in the muscles of CKD + LPD rats. Furthermore, KA supplementation reversed the elevation in p66Shc and FoxO3a expression in the muscles of CKD + LPD rats.

**Conclusions:**

Our results showed that KA supplementation to be beneficial to muscle atrophy in CKD + LPD, which might be associated with improvement of oxidative damage and mitochondrial dysfunction through suppression of p66Shc and FoxO3a.

## Background

Dietary protein restriction is one of the major components of therapy for patients with chronic kidney disease (CKD). It can minimise uremic symptoms and slow the progression of renal failure [1]. However, there has been increasing concern regarding the risk of subsequent development of malnutrition including muscle wasting as a result of restricted-protein diets. Ketoacids (KA), a nitrogen-free ketoanalogue, can reduce endogenous urea formation, toxic ions, and metabolic products in the CKD model [2]. Moreover, KA can provide a sufficient amount of essential amino acids to maintain nutritional status in CKD patients [3]. Therefore, it has been prescribed together with low-protein diets (LPD) to patients with advanced CKD. Despite the large number of studies on low-protein diets with ketoacids (LPD + KA) that have been performed to improve muscle atrophy in 5/6 nephrectomy and type 2 diabetic nephropathy rat models [4–6], the mechanism of its preventive effects on oxidative damage and mitochondrial dysfunction in skeletal muscle of CKD remains unclear.

Oxidative stress is involved in the pathogenesis of a number of chronic diseases, such as CKD, cancer, chronic heart failure, and diabetes mellitus. In the setting of CKD, oxidative damage is a known cause of muscle atrophy, which can contribute to muscle dysfunction and mark myofibrillar proteins for degradation [7]. The imbalance between increased production of reactive oxygen species (ROS) and limited antioxidant capacity can lead to severe damage on cellular components such as DNA, proteins, nucleic acids, and lipids in pathological conditions [8]. Hydrogen peroxide (H_2_O_2_) is a major component of ROS, generated during mitochondrial respiration, which induced mitochondrial dysfunction in skeletal muscle [9]. Concurrently, oxidants may stimulate the pathways of skeletal muscle protein degradation, such as the ubiquitin-proteasome system (UPS) [10]. Mammalian cells have a sophisticated system for scavenging ROS to non-toxic forms to defend cells against oxidative stress induced by high levels of ROS. This antioxidant defence system is composed of antioxidant enzymes such as superoxide dismutase (SOD) and catalase [11]. So far, there is evidence that ROS generation in mitochondria is critical for mitochondrial dysfunction and decreased mitochondrial content, which are controlled by regulating signal transduction, gene expression, and redox reaction [12, 13]. Among these, p66Shc has been shown to contribute to mitochondrial ROS (mtROS) production by sequestering electrons from the respiratory chain to regulate its redox function within mitochondria [14, 15]. In addition, FoxO3a is a member of the FoxO family of proteins, which has been implicated in initiating protein degradation during muscle atrophy [16]. It has been reported that FoxO3a reduces ROS generation by the transcriptional activation of SOD and catalase [17, 18]. However, the role of p66Shc and FoxO3a in mediating oxidative stress in the skeletal muscle of CKD has not been reported.

Mitochondrial dysfunction plays a pivotal role in the pathology of muscle atrophy induced by disuse, cancer, and ageing [19–21]. We have previously shown that mitochondrial dysfunction, characterised by mitochondrial loss and sedentary dynamics, plays a key role in CKD-induced muscle atrophy [22]. In addition, previous clinical and animal studies have shown that CKD disrupts mitochondrial morphology and oxidative capacity, which subsequently causes oxidative damage [23]. However, the role of oxidative stress and mitochondrial dysfunction in CKD-induced muscle atrophy is a controversial topic. In the present study, we aimed to investigate the role of ROS generation with p66Shc and FoxO3a signalling and antioxidant enzymes on skeletal muscle mitochondrial dysfunction in CKD rats. We hypothesised that LPD + KA protects against CKD-induced oxidative damage and mitochondrial dysfunction by mediating the p66Shc and FoxO3a signalling in the muscles of 5/6 nephrectomised rats.

## Methods

### Animal experiments

Sprague-Dawley male rats (obtained from the Experimental Animal Centre of the Southern Medical University, China, certification no. SCXK (Yue) 2006–0015) weighing 180–220 g were housed in a room at a constant temperature with a 12-h light–12-h dark cycle and were given free access to food and water. These rats were kept according to the guidelines of Care and Use of Laboratory Animals formulated by the Ministry of Science and Technology of China, and all experimental procedures were approved by the Ethics Committee of the Southern Medical University. Male rats were randomly assigned to either the 5/6 nephrectomy group or the sham-operated group. Each animal in the nephrectomy group underwent 5/6 nephrectomy by the ablation of two-thirds mass of the left kidneys and subsequent right unilateral nephrectomy after 1 week. In a sham-operated rat, a sham operation was performed. Then, 1 week after the operation, the 5/6 nephrectomy group was randomly separated and maintained on three different diets: a normal-protein diet (22% protein, NPD), a low-protein diet (6% protein, LPD) or a LPD supplemented with KA (5% protein and 1% KA, LPD + KA). The sham group with a normal-protein diet acted as the control. Each group included ten rats. These diets were fed to the groups for a period of 24 weeks. By the end of the study, one rat in NPD group had died, but all the other rats survived.

### Experimental diets

Rats were fed either an NPD, LPD or LPD + KA diet. KA was provided by Beijing Fresenius Kabi Pharmaceutical Company Limited. The KA composition was as follows (mg/630 mg): racemic keto isoleucine, 67 mg; ketones leucine, 101 mg; phenylalanine ketone, 68 mg; ketones valine, 86 mg; DL-methionine hydroxy, 59 mg; lysine acetate, 105 mg; threonine, 53 mg; tryptophan, 23 mg; histidine, 38 mg; tyrosine, 30 mg; total N, 36 mg. The three diets were formulated according to the American Institute of Nutrition for Rodent Diets, AIN-93, and all had the same energy content (15.7 kJ/g (3.8 kcal/g)), vitamins and minerals. The details of the composition of the three diets are as described in our previous study [4]. The animals in all groups had free access to food and water provided ad libitum.

### Grip power and running distance

Grip power was measured using a dynamometer for rats (ZH-YLS-13A, Anhui Zhenghua Biological Instrument Equipment Co., Ltd., Huaibei, China). A rat was put on a metal bar and pulled horizontally. The power of traction when the rat released the metal bar was defined as the grip power. Running distance was measured using a treadmill for mice (Yuyan Instrument Co., Ltd., Shanghai, China), in accordance with a previously described protocol [24]. Rats were made to run on the motor-driven treadmill until they were exhausted, which was defined as the point at which they remained on the electrical shocker plate (mild stimulation of 0.2 mA, equivalent to a medically used electric therapy equipment) at the end of the treadmill for more than 30 s. The treadmill was set at a 10% incline; the speed was 30 cm/s at the beginning and was increased by 3 cm/s every 2 min. The average running time until exhaustion was approximately 40 min.

### Biochemical parameters

After 24 weeks of treatment, the rats were sacrificed by sodium pentobarbital and blood samples were subsequently collected. Serum biochemical indexes serum creatinine (Scr), blood urea nitrogen (BUN) and albumin (ALB) were detected using a Roche automatic biochemical analyser.

### Morphological studies (HE, SDH staining) and measurement of myofiber size and SDH activity

The tibialis anterior (TA) muscle samples were sectioned and stained with haematoxylin and eosin (HE) and succinate dehydrogenase (SDH, complex II of the respiratory chain) in line with standard procedures. Myofiber cross-sectional area (CSA) and SDH activity were determined as previously reported [22].

### Assay of 26S proteasome activity

Chymotrypsin-like activity of proteasome was assayed using the fluorogenic peptide (LLVY-MCA, Enzo Life Sciences item #P802–0005) as described previously [25]. Assays were carried out in a microtiter plate by diluting 25 mg of cytosolic protein into 200 mL of 10 mM MOPS, pH 7.4 containing 25 mM LLVY-MCA (substrate), 25 mM ATP and 5.0 mM Mg^2+^. Rate of fluorescent product formation was measured with excitation and emission wavelengths of 350 and 440 nm, respectively. Peptidase activities were measured in the absence and presence (20 mM) of the proteasome-specific inhibitor epoxomicin and the difference between the two rates was attributed to the proteasome.

### Determination of malondialdehyde (MDA) activity and carbonyl content

A part of the homogenate was used for the determination of quadriceps muscle activity of MD, and was determined according to the method described by Buege and Aust [26]. The results were expressed as nanomoles per milligram protein. The other part of the homogenate was used for the determination of quadriceps muscle content of MDA by colorimetric method as previously described [27], using a protein carbonyl colorimetric assay kit. The result was expressed as nanomoles per milligram protein.

### Determination of lipid hydroperoxides

A modified ferrous oxidation-xylenol (FOX) orange technique was performed to measure the lipid hydroperoxides in accordance with a previously described protocol [28]. Briefly, quadriceps muscles were homogenised (1:4 wt/vol) in potassium phosphate buffer (50 mM, pH 7.8) and centrifuged at 12,000*g* for 15 min at 4 °C. For this assay, the pellet was discarded and 20 μl of the supernatant (250 μg of protein) was mixed with FOX reagent (250 μM ammonium ferrous sulphate, 100 μM xylenol orange and 25 mM H_2_SO_4_) at a final volume of 200 μl. It was incubated at room temperature for 30 min. The absorbance of the samples was read at 560 nm.

### Detection of ROS generation by dihydroethidium fluorescence staining

Dihydroethidium (DHE) oxidation products were detected as described previously [29]. Quadriceps muscles were cut into 10-μm-thick sections and were incubated with DHE (5 μM) in PBS in a light-protected incubator at 37 °C for 30 min. They were washed with PBS to remove excess DHE and then mounted. The fluorescence was evaluated in a confocal microscope (Zeiss LSM510Meta). Laser excitation at 488 nm and emission at 610 nm were used. The detection was made using a 560-nm long-pass filter. ImageJ (NIH) software was applied to quantitatively analyse the fluorescent images. The results were shown as arbitrary units of fluorescence.

### Muscle mitochondrial isolation

Mitochondrial isolation from the skeletal muscle was modified from the protocol described by Boutagy [30]. Briefly, red muscle was removed from the quadriceps muscle and finely minced with scissors. The muscle was then transferred to 10 ml mitochondrial homogenate buffer in tissue homogeniser. The muscle was homogenised using a motorised pestle and kept on ice at all times. After homogenisation, the homogenate buffer was gently poured into a 15-ml centrifuge tube and centrifuged at 1300 *g* for 5 min at 4 °C. The supernatant was absorbed onto a mitochondrial centrifugation buffer in an overspeed centrifuge tube and centrifuged at 17,000 *g* for 10 min at 4 °C. Soon after, the sediment was transferred into 9 ml of isolation buffer in another pre-chilled overspeed centrifuge tube. This was centrifuged at 10,000 *g* for 10 min at 4 °C. The sediment was transferred to a new pre-chilled 1.5-ml microcentrifuge tube and re-suspended in 1 ml of isolation buffer. This was centrifuged at 8000 *g* for 10 min at 4 °C. The supernatant was removed, and the sediment was gently mixed with 300 μl of storage buffer. The concentration of mitochondrial protein was determined with a BCA Protein Assay kit (Pierce, Rockford, IL, USA) and the protein yields of IFM were calculated accordingly.

### Oxygen consumption rate (OCR)

OCR was measured using the Seahorse XF^e^24 Extracellular Flux Analyzers (Seahorse Bioscience, Billerica, MA, USA), as described previously [31]. Ten micrograms of mitochondria (3 to 6 μl) were loaded at the centre of the XF24 cell culture microplates (Seahorse Bioscience) on ice, and 50 μl of the substrates (5 mM pyruvate plus 5 mM malate) and 440 μl of mitochondrial assay solution (MAS) (70 mM sucrose, 220 mM mannitol, 5 mM KH_2_PO_4_, 5 mM MgCl_2_, 2 mM HEPES, 1 mM EGTA, and 0.2% BSA, pH 7.4) were carefully added on top. All the chemicals loaded in the Seahorse cartridge ports were diluted in MAS (pH = 7.4).

### Measurement of electron transport chain activity

All assays were performed at 30 °C with a Shimadzu UV-1601 spectrophotometer. The specific activities of complexes I, II, III and IV were assayed as described by Sundaram Kumaran et al. [32].

### Determination of mitochondrial H_2_O_2_ content, SOD and catalase activity

The hydrogen peroxide content in the skeletal muscle mitochondria was measured by the colorimetric method as previously described [33], using a commercial kit, based on the reaction with molybdic acid (Jiancheng Biotech Inc., Nanjing, China). Adduct was measured spectrophotometrically at 405 nm in a plate reader (TECAN infinite M200, USA) in strict accordance with the manufacturer’s instructions. The total SOD activity (U/mg protein) in the mitochondria of the gastrocnemius muscles was measured using a commercial kit (Jiancheng Biotech Inc., Nanjing, China) in strict accordance with the manufacturer’s instructions. The adduct was measured spectrophotometrically at 550 nm with a plate reader (TECAN infinite M200, USA). Similarly, the activity of catalase (U/mg protein) in the mitochondria of the gastrocnemius muscles was measured using a commercial kit (Jiancheng Biotech Inc., Nanjing, China), which is based on the reaction of ammonium molybdate with H_2_O_2_ to form a light-yellow complex compound. Adduct was measured spectrophotometrically at 405 nm with a plate reader (TECAN infinite M200, USA) in strict accordance with the manufacturer’s instructions.

### Quantitative real-time PCR

Total RNA was isolated from the quadriceps muscles using Trizol (Invitrogen, Carlsbad, CA). RNA concentration and integrity were assessed. cDNA was synthesised using iScript cDNA Synthesis Kit at 70 °C for 10 min, followed by incubation at 42 °C for 60 min and at 95 °C for 10 min. The genes analysed were p66Shc, FoxO3a, Atrogin-1, MuRF-1, MUSA1, C5 and C2 proteasome subunits and GAPDH (reference gene) (Table 1). All primers were synthesised by Invitrogen. Quantitative real-time PCR was run for all genes separately, and amplifications were performed by the ABI Prism 5700 Sequence Detection System (Applied Biosystems) using SYBR Green PCR Master Mix (Applied Biosystems). Results were quantified as Ct values, where Ct is defined as the threshold cycle of the polymerase chain reaction at which the amplified product is first detected. Expression was normalised by GAPDH levels as an endogenous reference. Sham group levels were arbitrarily set at 1.

**Table 1:** Primer sequences

Gene	Forward	Reverse
P66SHC	5′-TACAACCCACTTCGGAATGGTCT-3′	5′-ATGTACCGAACCAAGTAGG-3′
FoxO3a	5′-CAGGTGTGTGCTGCTATGAACATC-3′	5′-GTCTTCGTGCTCGGTGATG-3′
C5 subunit	5′-GCTGCTCGACAACCAGGTTGGCTTC-3′	5′-CAGTGTACACATCCCTCTCGGCTGCAG-3′
C2 subunit	5′-TTGAAGAAAGACCACAGAGAAAAGCACAGC-3′	5′-GTATGCCCCTGCATCCTCATGTCCTC-3′
Atrogin-1	5′-TACTAAGGAGCGCCATGGATACT-3′	5′-GTTGAATCTTCTGGAATCCAG GAT-3′
MuRF1	5′-GTGTGAGGTGCCTACTTGCT-3′	5′-ACTCAGCTCCTCCTTCACCT-3′
MUSA1	5′-ACCACGACCCTGATGATGAGC-3′	5′-GGTCAGGCTCTTCCATTCGTCT-3′
GAPDH	5′-GTTCAACGGCACAGTCAAGG-3′	5′-GTGGTGAAGACGCCAGTAGA-3′

### Western blotting

Snap-frozen quadriceps muscle tissues were homogenised in lysis buffer as previously reported. Cytosolic and mitochondrial proteins were separated on a 10% SDS-PAGE gel and then transferred to a PVDF membrane (Bio-Rad Laboratories, Hercules, CA, USA). The membrane’s non-specific binding sites were blocked at 26 °C for 1 h with 5% non-fat milk powder in Tris-buffered saline/Tween-20 (TBST) and then incubated overnight at 4 °C with primary antibodies. After washing with TBST, the membranes were incubated with secondary antibodies for 1 h at room temperature with shaking. After washing, protein bands were detected and analysed using a ChemiDoc™ MP Imaging System (Bio-Rad Laboratories, CA, USA). VDAC and GAPDH were used as the loading controls for mitochondrial protein and cytosolic protein, respectively. Results were expressed as the integrated optical density relative to VDAC or GAPDH. SOD1 (1:1000, SAB2500976) was obtained from Sigma-Aldrich (Diegem, Belgium). VDAC (1:1000, #4661) and FoxO3a (1:1000, #2497) were obtained from Cell Signaling Technologies (Danvers, MA, USA). p66Shc (1:200, sc-1695) was obtained from Santa Cruz Biotechnology (CA, USA). GAPDH (1:1000, 60004-1-Ig) was obtained from Proteintech (Chicago, IL, USA).

### Statistical analysis

Results are shown as the mean ± SD. One-way analysis of variance (ANOVA) followed by the Student-Newman-Keuls test was used to compare the differences between the means in more than two groups. The level of significance was set at *P* < 0.05. All the statistical analyses were performed with SPSS 16.0 for Windows (SPSS Inc., Chicago, IL, USA).

## Results

### Ketoacid supplementation preserves renal function in CKD + LPD rats

At the end of the study, the 5/6 nephrectomy group displayed significantly higher Scr and BUN levels compared with the sham group. The level of Scr was found to be highest in the NPD group, but significantly decreased in the LPD group and lowest in the LPD + KA group. Meanwhile, BUN levels were highest in the NPD group and significantly reduced in the LPD group; the LPD + KA group had the lowest values. On the other hand, serum ALB levels were lower in the 5/6 nephrectomy groups than the sham group. Among the 5/6 nephrectomy groups, the LPD group had lower serum ALB levels than the NPD and LPD + KA groups, but no statistical differences were observed between the NPD and LPD + KA groups (Table 2).

**Table 2:** Renal function data (means ± SD)

Group	Scr(μmol/l)	BUN(mmol/l)	ALB(g/l)
Sham	60.61 ± 22.80	7.21 ± 2.12	89.40 ± 17.25
NPD	168.11 ± 43.18***	17.01 ± 3.32***	60.84 ± 12.21**
LPD	141.61 ± 36.67***^††^	12.01 ± 7.78**^†^	45.16 ± 10.89***^††^
LPD + KA	135.59 ± 25.66***^††‡^	9.08 ± 1.89*^††‡^	59.67 ± 5.66**^‡^

*Sham* normal-protein diet, *NPD* normal-protein diet, *LPD* low-protein diet, *LPD + KA* low-protein diet supplemented with KA, *Scr* serum creatinine *BUN* blood urea nitrogen, *ALB* albumin.

Mean value was significantly different from that of the sham group: *P < 0.05, **P < 0.01,***P < 0.001

Mean value was significantly different from that of the NPD group: ^†^*P* < 0.05, ^††^*P* < 0.01

Mean value was significantly different from that of the LPD group: ^‡^*P* < 0.05

### Ketoacid supplementation improves muscle atrophy and function in CKD + LPD rats

**Fig. 1:**
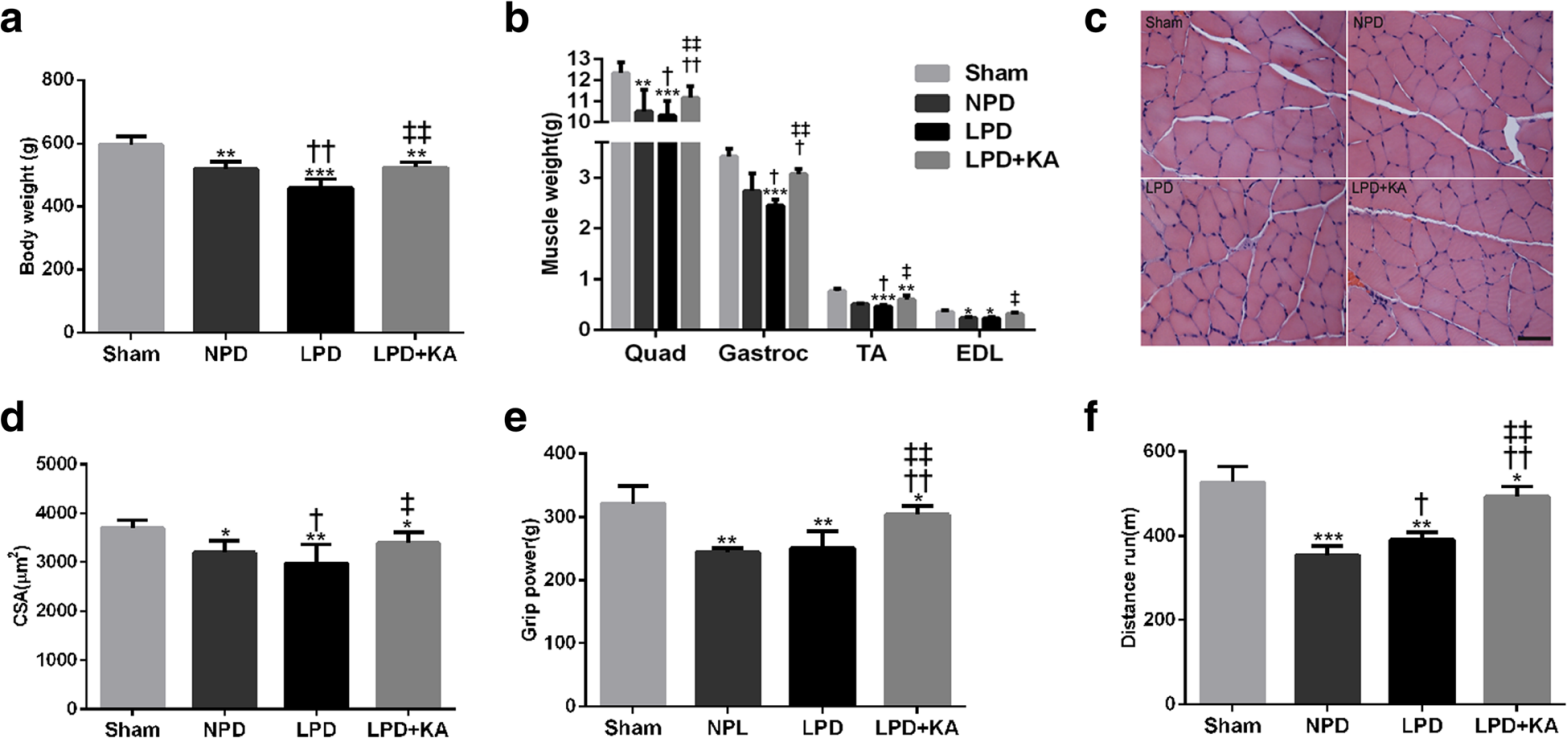
Body weight, muscle mass, muscle fibre cross-sectional area (CSA) and muscle function in the experimental groups. **a** Body weight changes. **b** Weights of quadriceps (Quad), gastrocnemius (Gastroc), tibialis anterior (TA) and extensor digitorum longus (EDL) muscles normalised by tibia length. **c** Cross sections of tibialis anterior (TA) muscle stained with haematoxylin and eosin (HE). Scale bar 50 μm. **d** Muscle fibre CSA (mm^2^) of TA muscle. **e** Grip power. **d** Running distance. Results are presented as the mean ± SD, *n* = 6 per group. Mean value was significantly different from that of the sham group: *P < 0.05, **P < 0.01. Mean value was significantly different from that of the NPD group: †*P* < 0.05, ††*P* < 0.01. Mean value was significantly different from that of the LPD group: ‡*P* < 0.05. Sham, normal-protein diet; NPD, normal-protein diet; LPD, low-protein diet; LPD + KA, low-protein diet supplemented with ketoacids

The body weight of the sham group was significantly higher than that of the 5/6 nephrectomy group. Among the 5/6 nephrectomy group, body weight was found to be the lowest in the LPD group and significantly increased in the LPD + KA group. No statistical differences were observed between the NPD and LPD + KA groups (Fig. 1a). Moreover, the quadriceps (Quad), gastrocnemius (Gastroc), tibialis anterior (TA) and extensor digitorum longus (EDL) muscle masses of the NPD and LPD groups were lower than those of the sham group. However, KA supplementation partially decreased the muscles mass losses compared to the LPD group (Fig. 1b). Furthermore, the improved muscle mass in the LPD + KA group was confirmed by an increase in the mean cross-sectional area of the TA muscle in the LPD group (Fig. 1c, d). The grip power was lower in the NPD and LPD group than in the sham group. However, the deceased grip power was partially corrected with KA supplementation in the LPD + KA group. There was no difference between the NPD and LPD groups (Fig. 1e). Similarly, the running distance of the NPD and LPD groups was significantly reduced, while KA supplementation triggered an increase compared to the LPD group. On the other hand, the LPD + KA group also showed an increase in the running distance compared to the NPD group (Fig. 1f).

### Ketoacid supplementation reduces the upregulation of genes related to the ubiquitin-proteasome system and 26S proteasome activity in the muscles of CKD + LPD rats

**Fig. 2:**
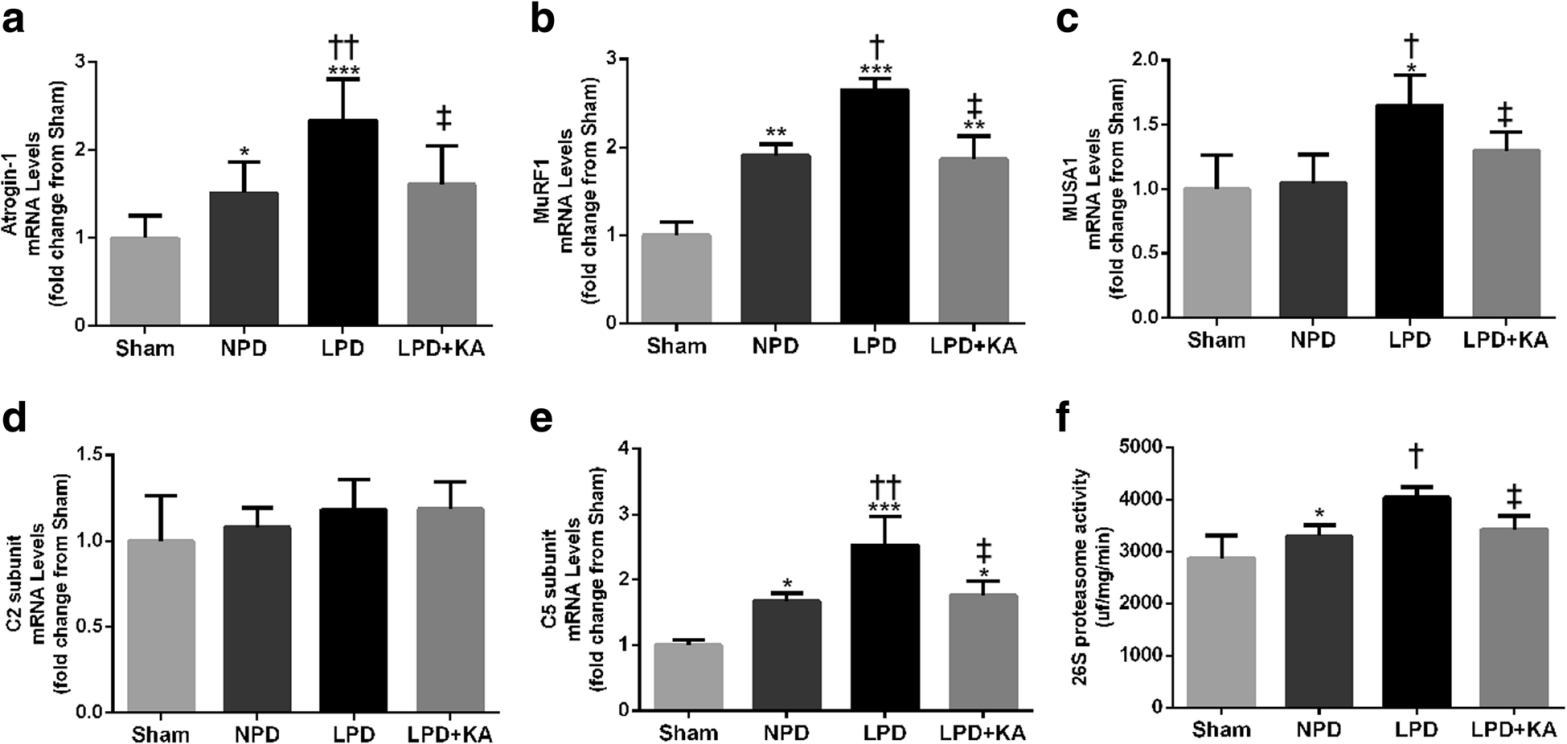
The ubiquitin-proteasome pathway in skeletal muscle of the experimental groups. **a** Atrogin-1, **b** MuRF1, **c** MUSA1, **d** C5 subunit and **e** C2 subunit mRNA levels, and **f** 26S chymotrypsin-like proteasome activity. Results are presented as the mean ± SD, *n* = 6 per group. Mean value was significantly different from that of the sham group: *P < 0.05, **P < 0.01. Mean value was significantly different from that of the NPD group: †*P* < 0.05, ††*P* < 0.01. Mean value was significantly different from that of the LPD group: ‡*P* < 0.05. Sham, normal-protein diet; NPD, normal-protein diet; LPD, low-protein diet; LPD + KA, low-protein diet supplemented with ketoacids

The 5/6 nephrectomy groups exhibited a significant induction of mRNA expression in Atrgin-1 and MuRF1 compared to the sham group; however, KA supplementation reduced the levels of Atrgin-1 and MuRF1 mRNA in the LPD + KA group compared to the LPD group (Fig. 2a, b). In addition, the LPD group displayed an increase in the expression of MUSA1 mRNA, and this change was abolished by KA intervention (Fig. 2c). Moreover, the levels of C5 proteasome subunit mRNA and 26S chymotrypsin-like proteasome activity were significantly increased in the NPD and LPD groups compared with the sham group, and this change of the LPD group was also abolished by KA supplementation (Fig. 2e, f). However, no changes were observed in C2 proteasome subunit mRNA levels among all the groups (Fig. 2d).

### Ketoacid supplementation suppresses oxidative stress in the muscles of CKD + LPD rats

**Fig. 3:**
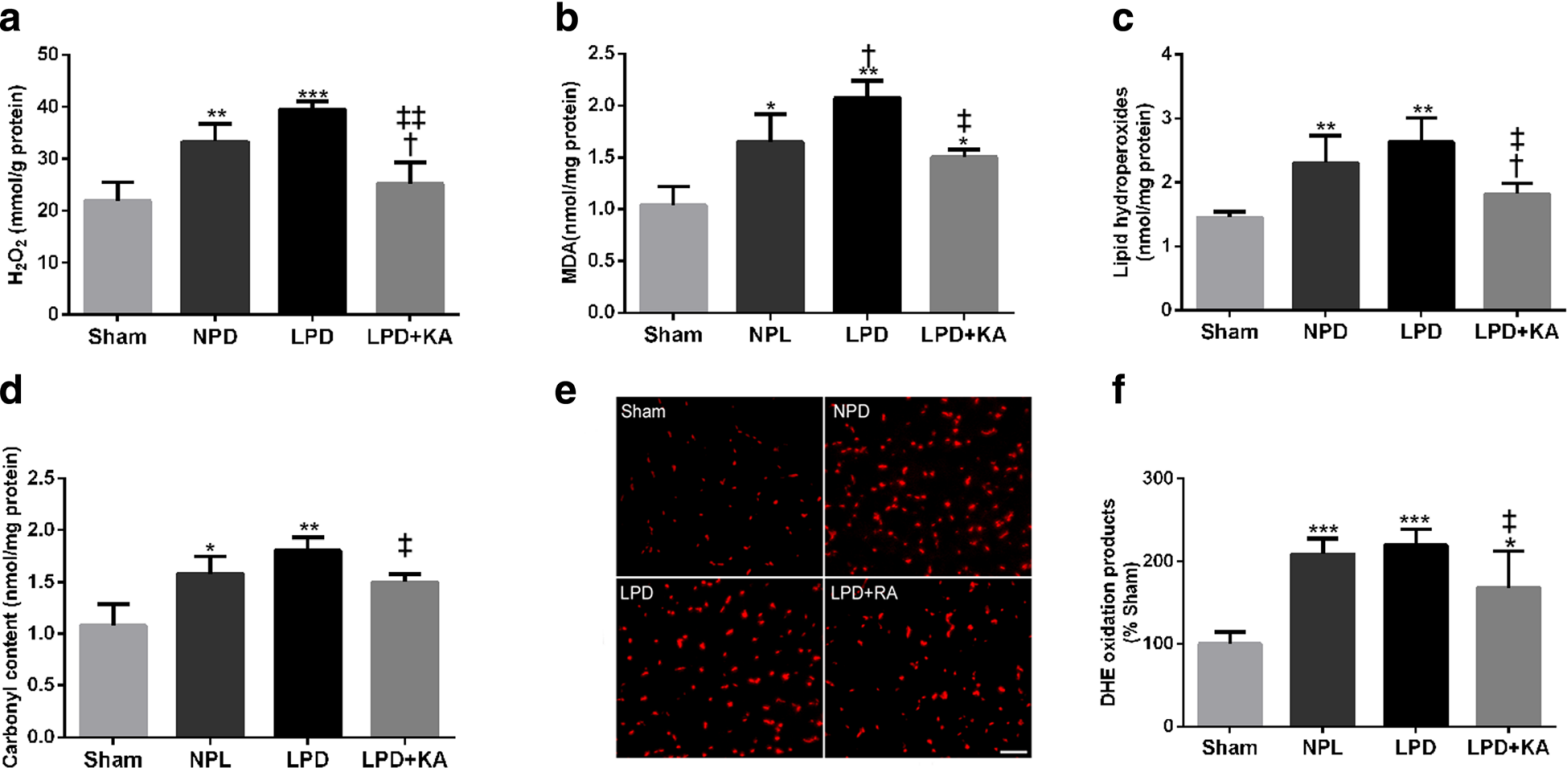
The marks of oxidative stress in skeletal muscle of the experimental groups. Quantification of skeletal muscle **a** H_2_O_2_, **b** MDA, **c** lipid hydroperoxides and **d** carbonyl contents. **e** Immunofluorescence staining for dihydroethidium (DHE). Scale bar = 100 μm. **f** Quantification of DHE intensity. Results are presented as the mean ± SD, *n* = 6 per group. Mean value was significantly different from that of the sham group: *P < 0.05, **P < 0.01. Mean value was significantly different from that of the NPD group: †*P* < 0.05, ††*P* < 0.01. Mean value was significantly different from that of the LPD group: ‡*P* < 0.05. Sham, normal-protein diet; NPD, normal-protein diet; LPD, low-protein diet; LPD + KA, low-protein diet supplemented with ketoacids

Muscle atrophy in CKD is associated with increased oxidative stress. Therefore, the markers of ROS, such as H_2_O_2_, MDA, lipid hydroperoxide, carbonyl content and DHE oxidation products, were detected. The NPD and LPD groups showed higher levels of H_2_O_2_ (Fig. 3a), MDA (Fig. 3b), lipid hydroperoxide (Fig. 3c), carbonyl content (Fig. 3d) and DHE oxidation products (Fig. 3e, f) in skeletal muscle compared to the sham group. Moreover, a low protein diet also caused a slight increase in the markers of ROS compared to the NPD group, although these changes were not statistically significant except for MDA levels. The addition of KA to LPD prevented the overexpression of these ROS markers.

### Ketoacid supplementation improves mitochondrial oxidative capacity in the muscles of CKD + LPD rats

**Fig. 4:**
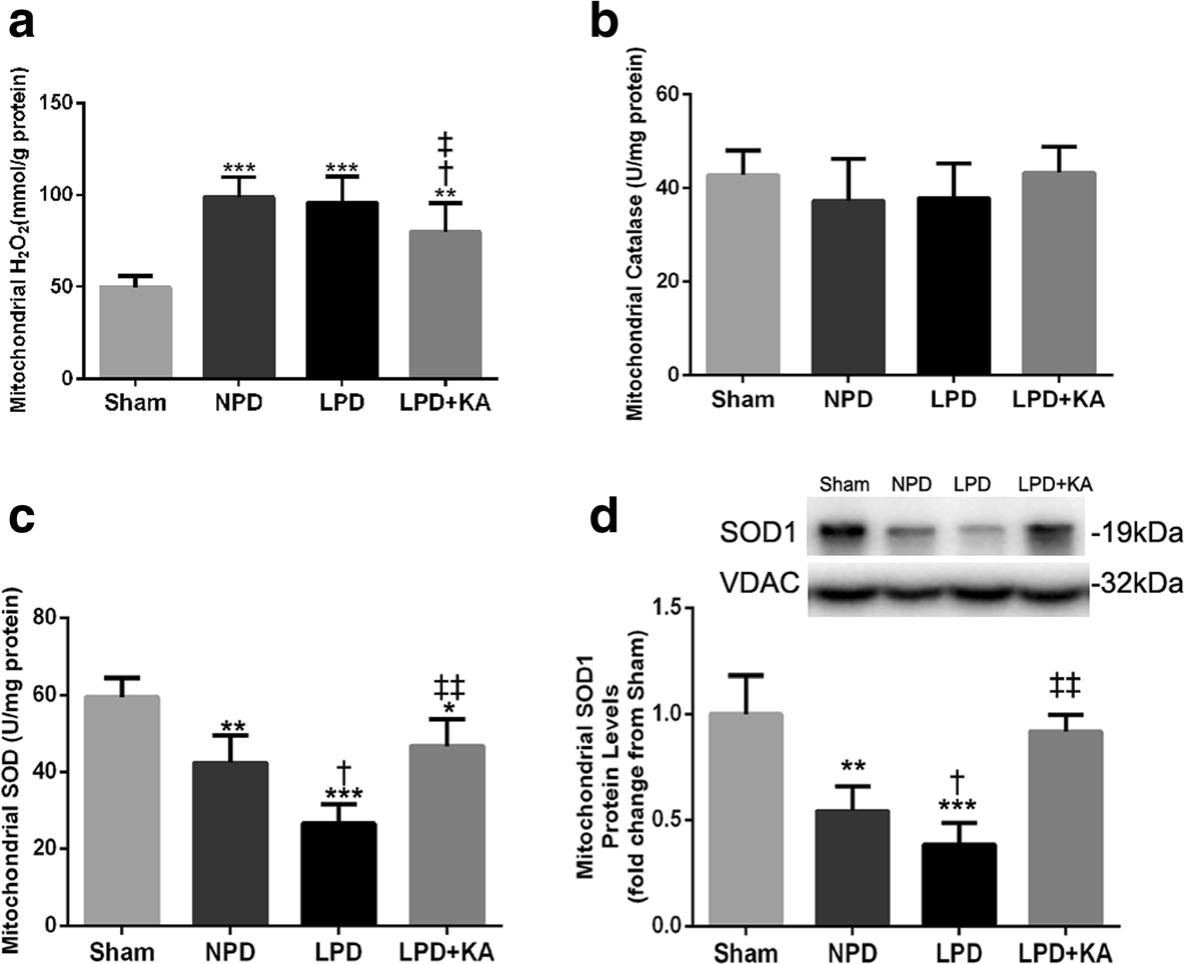
Mitochondrial oxidative capacity in skeletal muscle of the experimental groups. **a** Mitochondrial H_2_O_2_ content, **b** mitochondrial catalase activity, **c** mitochondrial SOD activity and **d** mitochondrial SOD1 protein. Results are presented as the mean ± SD, *n* = 6 per group. Mean value was significantly different from that of the sham group: *P < 0.05, **P < 0.01. Mean value was significantly different from that of the NPD group: †*P* < 0.05, ††*P* < 0.01. Mean value was significantly different from that of the LPD group: ‡*P* < 0.05. Sham, normal-protein diet; NPD, normal-protein diet; LPD, low-protein diet; LPD + KA, low-protein diet supplemented with ketoacids

To analyse the consequences of KA supplementation on mitochondrial oxidative capacity, we evaluated the levels of mitochondrial H_2_O_2_, catalase and SOD in the skeletal muscle of the experimental rats. The NPD and LPD groups displayed an increase in the mitochondrial H_2_O_2_ content compared with the sham group, while KA supplementation decreased the elevation of mitochondrial H_2_O_2_ compared to the LPD and NPD groups (Fig. 4a). Moreover, the mitochondrial catalase activity was slightly reduced in the NPD and LPD groups, and these changes appeared to be partially reversed with KA supplementation, as these differences did not reach significance (Fig. 4b). Furthermore, mitochondrial SOD activity and SOD1 protein were decreased in the NPD and LPD groups compared to those in the sham group. In addition, the LPD group showed a significant increase in mitochondrial SOD activity compared to the NPD group (Fig. 4c). Interestingly, the decreased mitochondrial SOD activity and SOD1 protein in the LPD group was prevented by KA supplementation (Fig. 4c, d).

### Ketoacid supplementation increases the activity of mitochondrial electron transport chain complexes in the muscles of CKD + LPD rats

**Fig. 5:**
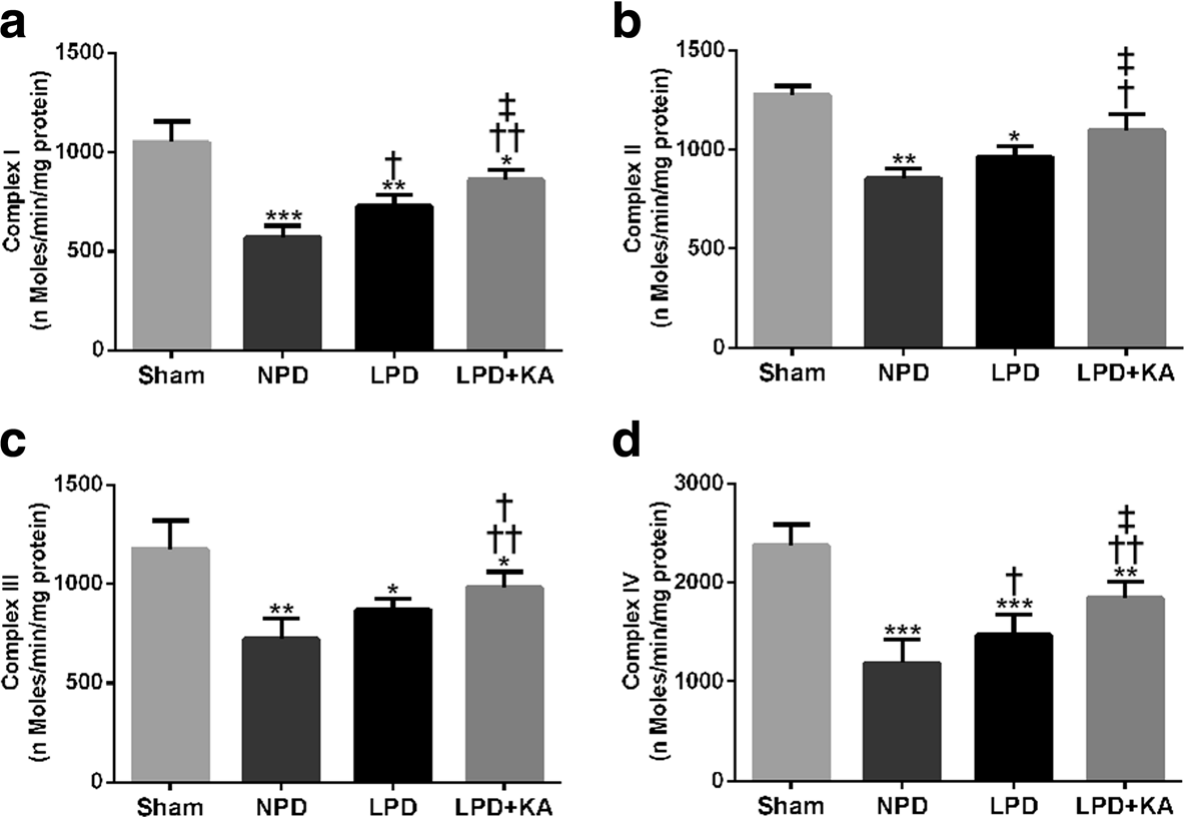
Activities of mitochondrial electron transport chain complexes in skeletal muscle of the experimental groups. The activity of **a** mitochondrial complex I (NADH-coenzyme Q oxidoreductase), **b** mitochondrial complex II (succinate dehydrogenase-coenzyme Q oxidoreductase), **c** mitochondrial complex III (coenzyme Q cytochrome c oxidoreductase) and **d** mitochondrial complex IV (cytochrome c oxidase) from skeletal muscle of the experimental rats. Results are presented as the mean ± SD, *n* = 6 per group. Mean value was significantly different from that of the sham group: *P < 0.05, **P < 0.01. Mean value was significantly different from that of the NPD group: †*P* < 0.05, ††*P* < 0.01. Mean value was significantly different from that of the LPD group: ‡*P* < 0.05. Sham, normal-protein diet; NPD, normal-protein diet; LPD, low-protein diet; LPD + KA, low-protein diet supplemented with ketoacids

To determine whether CKD induces mitochondrial dysfunction, we firstly determined the activities of mitochondrial electron transport chain complexes in gastrocnemius muscle. The activities of mitochondrial complexes I, II, III and IV were significantly decreased in the 5/6 nephrectomy groups compared to those in the sham group (Fig. 5a–d). Among the 5/6 nephrectomy groups, the activities of mitochondrial complexes I and IV were found to be the lowest in the NPD group, and significantly higher in the LPD and LPD + KA groups; however, the LPD + KA group showed a significantly higher level of activity compared to the LPD group (Fig. 5a, d). In addition, the NPD and LPD groups showed significantly lower levels of activity in mitochondrial complexes II and III compared to the sham group and were not statistically significant between the NPD and LPD groups. As expected, the LPD + KA group exhibited reduced levels of activity in mitochondrial complexes II and III compared to the LPD and NPD groups (Fig. 5b, c).

### Ketoacid supplementation improves the mitochondrial content and function in the muscles of CKD + LPD rats

**Fig. 6:**
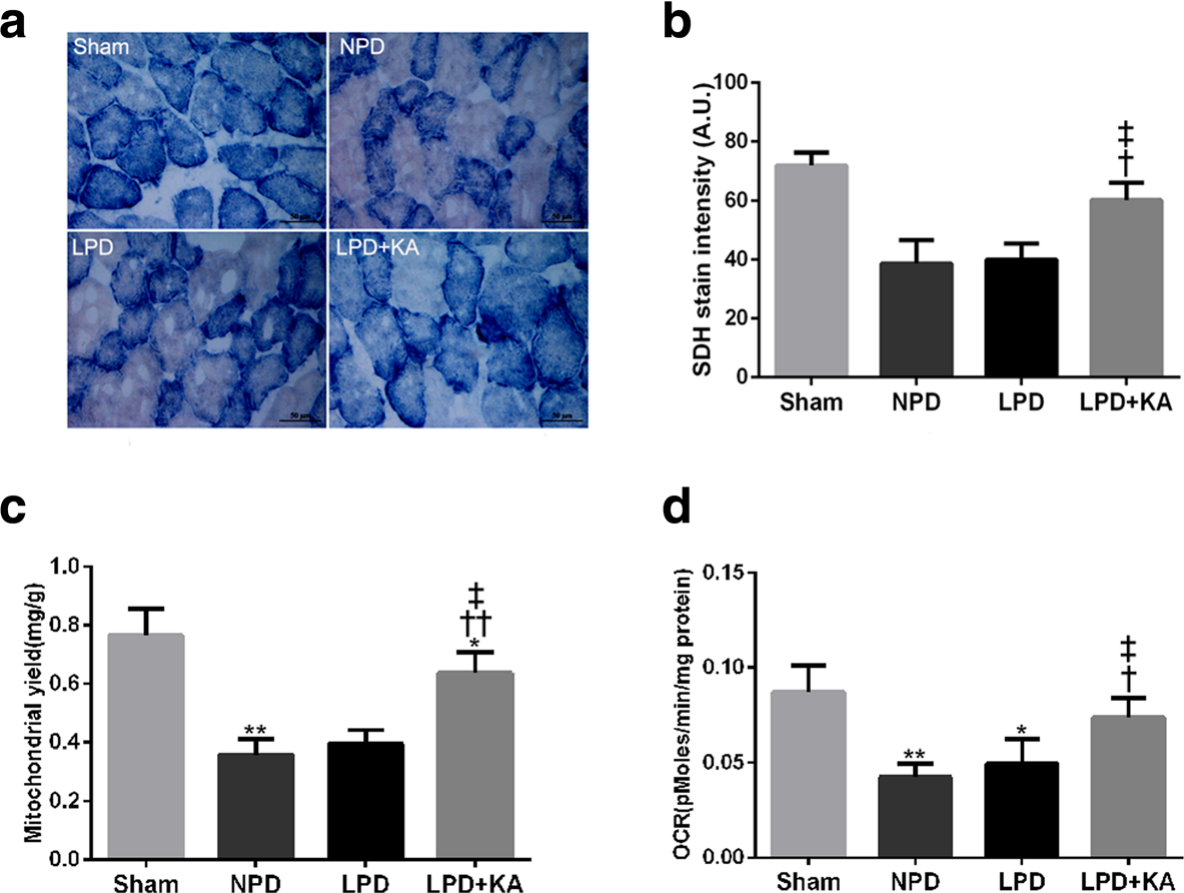
Mitochondrial content and mitochondrial oxygen consumption rate (OCR) in skeletal muscle of the experimental groups. **a** SDH staining was performed on 10-μm-thick sections from gastrocnemius muscles frozen in liquid nitrogen-cooled isopentane. Scale bar 50 μm. **b** Quantification of SDH-stain intensity (expressed in A.U). **c** Mitochondrial yield in isolated quadriceps muscles. **d** The mitochondrial oxygen consumption rate (OCR) in isolated quadriceps muscles. Results are presented as the mean ± SD, *n* = 6 per group. Mean value was significantly different from that of the sham group: *P < 0.05, **P < 0.01. Mean value was significantly different from that of the NPD group: †*P* < 0.05, ††*P* < 0.01. Mean value was significantly different from that of the LPD group: ‡*P* < 0.05. Sham, normal-protein diet; NPD, normal-protein diet; LPD, low-protein diet; LPD + KA, low-protein diet supplemented with ketoacids

Mitochondrial content was assessed using SDH staining of TA muscles (Fig. 6a). The SDH stain intensity was markedly reduced in the NPD and LPD groups compared to that in the sham group, but distinctly increased in the LPD + KA group compared to that in the LPD group (Fig. 6b). Moreover, the results showed that the mitochondrial yield of intermyofibrillar mitochondria (IFM) was significantly lower in the NPD and LPD groups compared to that in the sham group, but showed remarkable improvement with KA treatment in the LPD + KA group compared to that in the LPD group (Fig. 6c). Consistently, the basal mitochondrial respiration ability (oxygen consumption rate, OCR) of IFM was decreased in the NPD and LPD groups compared to that in the sham group. Furthermore, the increase in OCR was also more prominent in the LPD + KA group than in the LPD group (Fig. 6d).

### Ketoacid supplementation decreases expression of p66Shc and FoxO3a proteins in the muscles of CKD + LPD rats

**Fig. 7:**
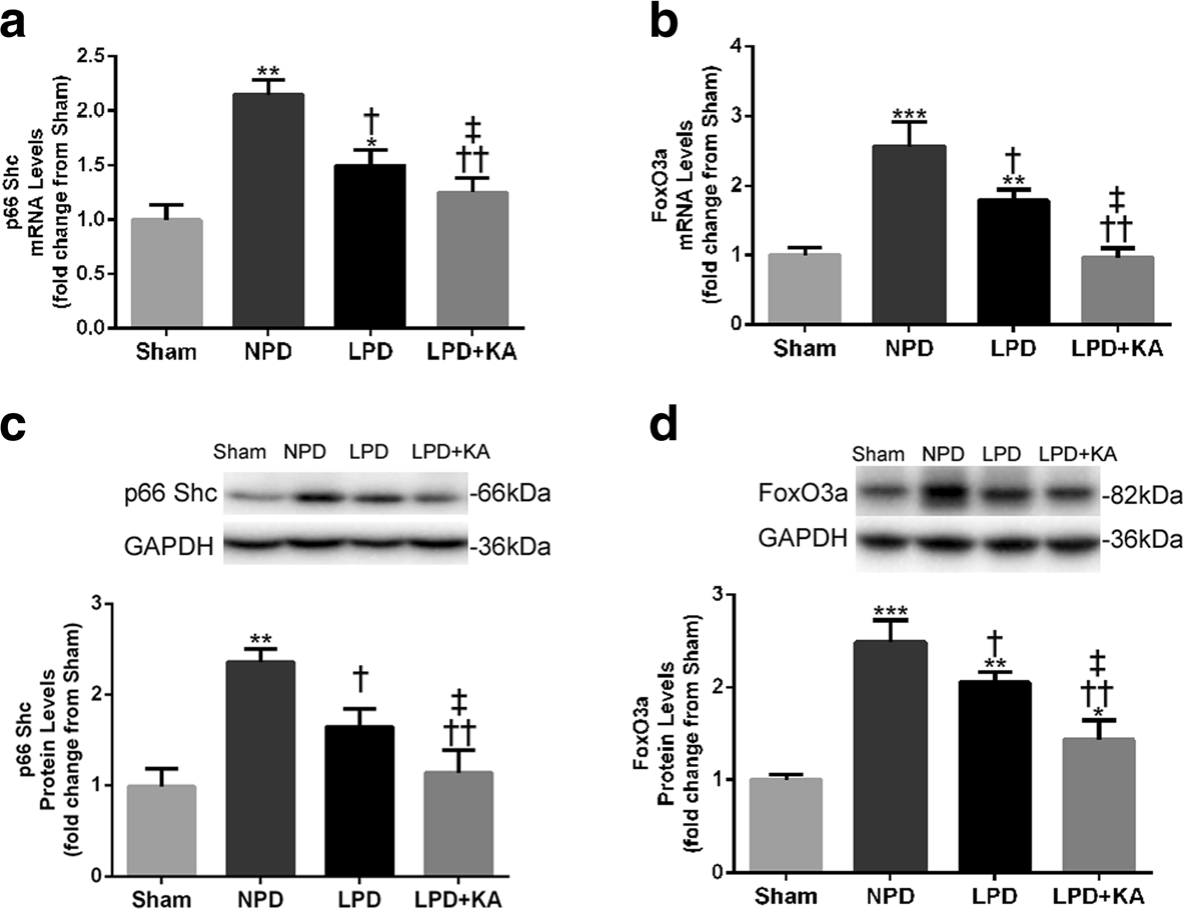
Expression of p66Shc and FoxO3a mRNA, and proteins in the skeletal muscle of the experimental groups. **a** p66Shc and **b** FoxO3a mRNA expression was measured by RT-PCR and is presented as corrected for GAPDH and normalised to the sham group. **c** Upper: representative immunoblotting of p66Shc and GAPDH. Lower: the ratio of p66Shc and GAPDH normalised to the sham group. **d** Upper: representative immunoblotting of FoxO3a and GAPDH. Lower: the ratio of FoxO3a and GAPDH normalised to the sham group. Results are presented as the mean ± SD, *n* = 6 per group. Mean value was significantly different from that of the sham group: *P < 0.05, **P < 0.01. Mean value was significantly different from that of the NPD group: †*P* < 0.05, ††*P* < 0.01. Mean value was significantly different from that of the LPD group: ‡*P* < 0.05. Sham, normal-protein diet; NPD, normal-protein diet; LPD, low-protein diet; LPD + KA, low-protein diet supplemented with ketoacids

Evidence indicates that p66shc serves as a redox enzyme and has been implicated in mitochondrial ROS generation and translation of oxidative signals. The expression of p66Shc protein and mRNA was significantly higher in the NPD and LPD groups, respectively, compared to that in the sham group. However, KA treatment decreased the level of the p66Shc protein and mRNA compared to the level in the LPD group. Moreover, LPD and LPD + KA groups displayed a lower level of p66Shc protein and mRNA than the NPD group (Fig. 7a, c). On the other hand, an upward trend in FoxO3a protein expression and mRNA in the NPD and LPD groups was also observed in comparison to the sham group, but KA supplementation induced a decrease compared to the LPD group. In addition, the LPD and LPD + KA groups also showed a lower level of the FoxO3a protein and mRNA than the NPD group (Fig. 7b, d).

## Discussion

These experiments provide novel insights into the mechanisms responsible for KA supplementation playing a protective role in muscle atrophy and its function in CKD-LPD rats. In the present study, the data indicate that CKD-LPD-induced loss of muscle mass and function is attributed to a significant reduction in fibre CSA compared to sham rats. Surprisingly, these changes were reversed by KA supplementation. Our findings are in agreement with our own previous studies as well as those conducted by other researchers [4–6], highlighting CKD-LPD-dependent fibre atrophy as the primary cause of muscle mass loss in advancing CKD [34]. Moreover, our findings confirm that KA supplementation suppresses UPS activation and protects skeletal muscle from oxidative damage in CKD + LPD rats. Furthermore, our results show that KA supplementation prevents decreases in the activity of mitochondrial electron transport chain complexes and increases mitochondrial respiration and content in the muscles of CKD + LPD rats. Furthermore, our findings confirm that KA supplementation reduces the production of mtROS, and p66Shc and FoxO3a expression in the muscles of CKD + LPD rats.

It is widely accepted that the UPS is the main route by which proteins are degraded during muscle atrophy. This involves the targeted degradation of proteins via modification by ubiquitin and subsequent proteolysis by the 26S proteasome [7]. Proteins targeted by ubiquitin are modified through the actions of three types of ubiquitin-conjugating enzymes: E1, E2 and E3. Three E3 proteins appear to mediate in skeletal muscle catabolism: Atrogin1/MAFbx, MuRF1 and MUSA1. Atrogin1 and MuRF1 are upregulated in a number of catabolic conditions including cancer, diabetes, kidney failure and sepsis [35]. Indeed, the expression of Atrogin-1, MuRF1 and MUSA1 were increased in CKD-LPD rats. Moreover, these increases correlate with increased C5 subunit mRNA and 26S proteasome activity. Importantly, these changes were prevented by KA supplementation, which is consistent with our results from previous works [4]. Collectively, the present results indicate that KA supplementation plays a muscle-protective role in CKD-LPD, at least in part, via inhibition of the UPS.

Considerable evidence has indicated that muscle atrophy with CKD has been linked to an altered oxidative status of redox-sensitive proteins [36], and increased oxidative modifications of virtually all cellular macromolecules, including lipids, DNA and proteins [7]. Muscle atrophy can be exacerbated by oxidative stress, which promotes the production of reactive carbonyl compounds and lipoperoxides leading to the accumulation of advanced glycation and lipoxidation end products [37]. In the present study, our data showed that CKD-LPD-induced loss of muscle mass and function is associated with increased oxidative damage including an increase in H_2_O_2_ and MDA levels, accumulation of carbonyl content and DHE oxidation products and increased levels of lipid peroxidation, suggesting that changes in redox homeostasis toward an oxidised state may be a contributor to skeletal muscle atrophy. Previous work from our own group [4] and others [5, 6] have shown that KA supplementation has a beneficial anti-atrophy effect in CKD animals, which is consistent with our results. Furthermore, we found that the marks of oxidative stress were decreased in skeletal muscle when supplemented with KA compared to LPD alone. Genetic manipulations of redox regulatory systems were found to modify the muscle atrophy process [37]. Collectively, the present results indicate that KA supplementation ameliorates oxidative damage in the skeletal muscle of CKD + LPD rats.

Skeletal muscle has a high mitochondrial content, and skeletal muscle mitochondria have been reported to exhibit an increase in mtROS in disuse [38] and ageing [39, 40]. In the present study, we have shown that isolated skeletal muscle mitochondria from CKD + LPD rats exhibit an increase in H_2_O_2_ generation, which was consistent with the role of mitochondria as a contributor to CKD-related muscle oxidative damage [41]. Moreover, treatment with KA protects against CKD-LPD-induced increases in mitochondrial H_2_O_2_ content, which may selectively protect mitochondria from oxidative damage. On the other hand, increased oxidative stress arises from an imbalance between pro-oxidant and antioxidant factors and is depicted in skeletal muscle under catabolic or dysfunctional conditions [42]. In the present study, our results show that the CKD-LPD group displays a decrease in mitochondrial catalase activity, SOD activity, and protein expression in skeletal muscle, which was reversed by KA supplementation. Importantly, while mitochondria-targeted antioxidant ameliorates muscle loss and mitochondrial dysfunction of skeletal muscle in ageing rats [39], targeted overexpression of mitochondrial catalase protects against cancer chemotherapy-induced skeletal muscle atrophy and dysfunction [43]. Therefore, strong evidence of redox imbalance-induced skeletal muscle atrophy supports our hypothesis that mitochondrial oxidative damage is a major determinant of skeletal muscle loss in CKD. In addition, KA treatment reduces mitochondrial ROS and provides clear antioxidant protective effects on muscle atrophy in CKD + LPD rats.

Skeletal muscle atrophy with CKD is associated with mitochondrial dysfunction including the decrease of activity in mitochondrial electron transport chain enzyme complexes and mitochondrial content, and reduced mitochondrial respiratory capacity [41, 44]. The mitochondrial respiratory chain is a powerful source of ROS, considered as a potential mechanism contributing to mitochondrial dysfunction. Although cumulative oxidative damage has been suggested to induce CKD-associated decline in mitochondrial function [44], the effect of mitochondrial dysfunction and mtROS as the underlying key regulators of the CKD-related atrophy process remains unclear. The present study shows that the activities of mitochondrial complexes I, II, III and IV were found to be significantly decreased in the skeletal muscle of CKD-LPD rats. To our surprise, KA supplementation improved the activity of complexes I, II, III and IV in the skeletal muscle of CKD-LPD rats. Several studies suggest that oxidative damage to mitochondrial DNA may be responsible for the decrease in the activity of electron transport chain enzyme complexes in aged rats and amyotrophic lateral sclerosis mouse models [45, 46]. Oxidative damage to proteins is associated with numerous alterations in mitochondrial respiratory capacity and amount. In the present study, we found that the mitochondrial content and the rate of oxygen consumption were significantly decreased in the skeletal muscle of CKD-LPD rats. Furthermore, KA supplementation can effectively reverse mitochondrial respiratory capacity and mitochondrial loss in the LPD + KA group. Evidence suggests that mitochondrial dysfunction plays a key role in the pathology of muscle atrophy induced by CKD [44, 47]. Ourselves as well as other researchers have previously reported that CKD-induced dominant mitochondrial dysfunction, characterised by mitochondrial loss, compromised mitochondrial respiration, and disrupted mitochondrial distribution and morphology [22, 47]. Collectively, the present results indicate that KA supplementation increases muscle mitochondrial mass and the activity of mitochondrial electron transport chain enzyme complexes and improves mitochondrial respiration in CKD + LPD rats.

Following these findings, we investigated several intracellular signalling pathways mediating mitochondrial ROS that could contribute to muscle wasting. Specifically, we focused on p66Shc and FoxO3a activation because both are activated by oxidant stress and both contribute to muscle wasting [48, 49]. Recent studies also implicate p66Shc in a redox-dependent pathway that sensitises cells to proapoptotic stimuli by activating AKT, phosphorylating FoxO transcription factors and preventing the induction of antioxidant/free radical scavenging genes [50]. Our findings confirm that upregulation of p66Shc and FoxO3a expression in the skeletal muscle of CKD-LPD rats and, importantly, treatment with KA decreases the expression of both p66Shc and FoxO3a in CKD + LPD rats. These results strongly suggest that the p66Shc-FoxO3a pathway plays a role in the regulation of mitochondrial ROS production and muscle oxidative stress responses, and that this pathway may mediate the anti-oxidative effects of KA.

## Conclusions

Our study demonstrated that CKD-LPD causes an increase in oxidative stress and mitochondrial damage in skeletal muscle, which may be associated with the upregulation of p66Shc and FoxO3a. KA supplementation plays a protective role in muscle atrophy in CKD-LPD rats. The effect may be mediated by KA-ameliorating UPS activation, oxidative stress injury, mitochondrial damage, and decreasing the expression of p66Shc and FoxO3a in the muscles of CKD-LPD rats. In addition, KA supplementation improves mitochondrial respiration and content and increases the activity of mitochondrial electron transport chain enzyme complexes in the muscles of CKD-LPD rats. Thus, these findings may provide relevant preclinical data for the use of LPD + KA in patients with CKD.

## References

[1] Fouque D, Wang P, Laville M, Boissel JP (2000). Low protein diets delay end-stage renal disease in non-diabetic adults with chronic renal failure. Nephrol Dial Transplant.

[2] Gao X, Wu J, Dong Z, Hua C, Hu H, Mei C (2010). A low-protein diet supplemented with ketoacids plays a more protective role against oxidative stress of rat kidney tissue with 5/6 nephrectomy than a low-protein diet alone. Br J Nutr.

[3] Feiten SF, Draibe SA, Watanabe R, Duenhas MR, Baxmann AC, Nerbass FB, Cuppari L (2005). Short-term effects of a very-low-protein diet supplemented with ketoacids in nondialyzed chronic kidney disease patients. Eur J Clin Nutr.

[4] Wang DT, Lu L, Shi Y, Geng ZB, Yin Y, Wang M, Wei LB (2014). Supplementation of ketoacids contributes to the up-regulation of the Wnt7a/Akt/p70S6K pathway and the down-regulation of apoptotic and ubiquitin-proteasome systems in the muscle of 5/6 nephrectomised rats. Br J Nutr.

[5] Huang J, Wang J, Gu L, Bao J, Yin J, Tang Z, Wang L, Yuan W (2013). Effect of a low-protein diet supplemented with ketoacids on skeletal muscle atrophy and autophagy in rats with type 2 diabetic nephropathy. PLoS One.

[6] Zhang YY, Huang J, Yang M, Gu LJ, Ji JY, Wang LJ, Yuan WJ (2015). Effect of a low-protein diet supplemented with keto-acids on autophagy and inflammation in 5/6 nephrectomized rats. Biosci Rep.

[7] Moylan JS, Reid MB (2007). Oxidative stress, chronic disease, and muscle wasting. Muscle Nerve.

[8] Sener G, Paskaloglu K, Satiroglu H, Alican I, Kacmaz A, Sakarcan A (2004). L-carnitine ameliorates oxidative damage due to chronic renal failure in rats. J Cardiovasc Pharmacol.

[9] Min K, Kwon OS, Smuder AJ, Wiggs MP, Sollanek KJ, Christou DD, Yoo JK, Hwang MH, Szeto HH, Kavazis AN (2015). Increased mitochondrial emission of reactive oxygen species and calpain activation are required for doxorubicin-induced cardiac and skeletal muscle myopathy. J Physiol.

[10] Li YP, Chen Y, Li AS, Reid MB (2003). Hydrogen peroxide stimulates ubiquitin-conjugating activity and expression of genes for specific E2 and E3 proteins in skeletal muscle myotubes. Am J Physiol Cell Physiol.

[11] Vendelbo MH, Nair KS (2011). Mitochondrial longevity pathways. Biochim Biophys Acta.

[12] Jackson MJ (2005). Reactive oxygen species and redox-regulation of skeletal muscle adaptations to exercise. Philos Trans R Soc Lond Ser B Biol Sci.

[13] Liu J, Peng Y, Feng Z, Shi W, Qu L, Li Y, Liu J, Long J (2014). Reloading functionally ameliorates disuse-induced muscle atrophy by reversing mitochondrial dysfunction, and similar benefits are gained by administering a combination of mitochondrial nutrients. Free Radic Biol Med.

[14] Pani G, Galeotti T (2011). Role of MnSOD and p66shc in mitochondrial response to p53. Antioxid Redox Signal.

[15] Gertz M, Fischer F, Leipelt M, Wolters D, Steegborn C (2009). Identification of Peroxiredoxin 1 as a novel interaction partner for the lifespan regulator protein p66Shc. Aging (Albany NY).

[16] Sandri M, Sandri C, Gilbert A, Skurk C, Calabria E, Picard A, Walsh K, Schiaffino S, Lecker SH, Goldberg AL (2004). Foxo transcription factors induce the atrophy-related ubiquitin ligase atrogin-1 and cause skeletal muscle atrophy. Cell.

[17] Huang C, Lin Y, Su H, Ye D (2015). Forsythiaside protects against hydrogen peroxide-induced oxidative stress and apoptosis in PC12 cell. Neurochem Res.

[18] Tan WQ, Wang K, Lv DY, Li PF (2008). Foxo3a inhibits cardiomyocyte hypertrophy through transactivating catalase. J Biol Chem.

[19] Joseph AM, Adhihetty PJ, Leeuwenburgh C (2016). Beneficial effects of exercise on age-related mitochondrial dysfunction and oxidative stress in skeletal muscle. J Physiol.

[20] Argiles JM, Lopez-Soriano FJ, Busquets S (2015). Muscle wasting in cancer: the role of mitochondria. Curr Opin Clin Nutr Metab Care.

[21] Calvani R, Joseph AM, Adhihetty PJ, Miccheli A, Bossola M, Leeuwenburgh C, Bernabei R, Marzetti E (2013). Mitochondrial pathways in sarcopenia of aging and disuse muscle atrophy. Biol Chem.

[22] Wang D, Chen J, Liu X, Zheng P, Song G, Yi T, Li S (2017). A Chinese herbal formula, Jian-Pi-Yi-Shen decoction, improves muscle atrophy via regulating mitochondrial quality control process in 5/6 nephrectomised rats. Sci Rep.

[23] Avin KG, Chen NX, Organ JM, Zarse C, O'Neill K, Conway RG, Konrad RJ, Bacallao RL, Allen MR, Moe SM (2016). Skeletal Muscle Regeneration and Oxidative Stress Are Altered in Chronic Kidney Disease. PLoS One.

[24] Tamaki M, Miyashita K, Wakino S, Mitsuishi M, Hayashi K, Itoh H (2014). Chronic kidney disease reduces muscle mitochondria and exercise endurance and its exacerbation by dietary protein through inactivation of pyruvate dehydrogenase. Kidney Int.

[25] Cunha TF, Bacurau AV, Moreira JB, Paixao NA, Campos JC, Ferreira JC, Leal ML, Negrao CE, Moriscot AS, Wisloff U (2012). Exercise training prevents oxidative stress and ubiquitin-proteasome system overactivity and reverse skeletal muscle atrophy in heart failure. PLoS One.

[26] Buege JA, Aust SD (1978). Microsomal lipid peroxidation. Methods Enzymol.

[27] Reznick AZ, Packer L (1994). Oxidative damage to proteins: spectrophotometric method for carbonyl assay. Methods Enzymol.

[28] Nourooz-Zadeh J, Tajaddini-Sarmadi J, Wolff SP (1994). Measurement of plasma hydroperoxide concentrations by the ferrous oxidation-xylenol orange assay in conjunction with triphenylphosphine. Anal Biochem.

[29] Liberman M, Bassi E, Martinatti MK, Lario FC, Wosniak J, Pomerantzeff PM, Laurindo FR (2008). Oxidant generation predominates around calcifying foci and enhances progression of aortic valve calcification. Arterioscler Thromb Vasc Biol.

[30] Boutagy NE, Pyne E, Rogers GW, Ali M, Hulver MW, Frisard MI (2015). Isolation of Mitochondria from Minimal Quantities of Mouse Skeletal Muscle for High Throughput Microplate Respiratory Measurements. J Vis Exp.

[31] Sahin E, Colla S, Liesa M, Moslehi J, Muller FL, Guo M, Cooper M, Kotton D, Fabian AJ, Walkey C (2011). Telomere dysfunction induces metabolic and mitochondrial compromise. Nature.

[32] Kumaran S, Subathra M, Balu M, Panneerselvam C (2004). Age-associated decreased activities of mitochondrial electron transport chain complexes in heart and skeletal muscle: role of L-carnitine. Chem Biol Interact.

[33] Molnar AM, Servais S, Guichardant M, Lagarde M, Macedo DV, Pereira-Da-Silva L, Sibille B, Favier R (2006). Mitochondrial H2O2 production is reduced with acute and chronic eccentric exercise in rat skeletal muscle. Antioxid Redox Signal.

[34] Cianciaruso B, Bellizzi V, Brunori G, Cupisti A, Filippini A, Oldrizzi L, Quintaliani G, Santoro D (2008). [Low-protein diet in Italy today: the conclusions of the Working Group from the Italian Society of Nephrology]. G Ital Nefrol.

[35] Bonaldo P, Sandri M (2013). Cellular and molecular mechanisms of muscle atrophy. Dis Model Mech.

[36] Beetham KS, Howden EJ, Small DM, Briskey DR, Rossi M, Isbel N, Coombes JS (2015). Oxidative stress contributes to muscle atrophy in chronic kidney disease patients. Redox Rep.

[37] Choi MH, Ow JR, Yang ND, Taneja R (2016). Oxidative Stress-Mediated Skeletal Muscle Degeneration: Molecules, Mechanisms, and Therapies. Oxidative Med Cell Longev.

[38] Talbert EE, Smuder AJ, Min K, Kwon OS, Szeto HH, Powers SK (2013). Immobilization-induced activation of key proteolytic systems in skeletal muscles is prevented by a mitochondria-targeted antioxidant. J Appl Physiol.

[39] Javadov S, Jang S, Rodriguez-Reyes N, Rodriguez-Zayas AE, Soto Hernandez J, Krainz T, Wipf P, Frontera W (2015). Mitochondria-targeted antioxidant preserves contractile properties and mitochondrial function of skeletal muscle in aged rats. Oncotarget.

[40] Chabi B, Ljubicic V, Menzies KJ, Huang JH, Saleem A, Hood DA (2008). Mitochondrial function and apoptotic susceptibility in aging skeletal muscle. Aging Cell.

[41] Yazdi PG, Moradi H, Yang JY, Wang PH, Vaziri ND (2013). Skeletal muscle mitochondrial depletion and dysfunction in chronic kidney disease. Int J Clin Exp Med.

[42] Powers SK, Morton AB, Ahn B, Smuder AJ (2016). Redox control of skeletal muscle atrophy. Free Radic Biol Med.

[43] Gilliam LA, Lark DS, Reese LR, Torres MJ, Ryan TE, Lin CT, Cathey BL, Neufer PD (2016). Targeted overexpression of mitochondrial catalase protects against cancer chemotherapy-induced skeletal muscle dysfunction. Am J Physiol Endocrinol Metab.

[44] Su Z, Klein JD, Du J, Franch HA, Zhang L, Hassounah F, Hudson MB, Wang XH (2017). Chronic kidney disease induces autophagy leading to dysfunction of mitochondria in skeletal muscle. Am J Physiol Renal Physiol.

[45] Sakellariou GK, Pearson T, Lightfoot AP, Nye GA, Wells N, Giakoumaki II, Griffiths RD, McArdle A, Jackson MJ (2016). Long-term administration of the mitochondria-targeted antioxidant mitoquinone mesylate fails to attenuate age-related oxidative damage or rescue the loss of muscle mass and function associated with aging of skeletal muscle. FASEB J.

[46] Jung C, Higgins CM, Xu Z (2002). Mitochondrial electron transport chain complex dysfunction in a transgenic mouse model for amyotrophic lateral sclerosis. J Neurochem.

[47] Balakrishnan VS, Rao M, Menon V, Gordon PL, Pilichowska M, Castaneda F, Castaneda-Sceppa C (2010). Resistance training increases muscle mitochondrial biogenesis in patients with chronic kidney disease. Clin J Am Soc Nephrol.

[48] Whidden MA, Smuder AJ, Wu M, Hudson MB, Nelson WB, Powers SK (2010). Oxidative stress is required for mechanical ventilation-induced protease activation in the diaphragm. J Appl Physiol (1985).

[49] Powers SK, Smuder AJ, Criswell DS (2011). Mechanistic links between oxidative stress and disuse muscle atrophy. Antioxid Redox Signal.

[50] Guo J, Gertsberg Z, Ozgen N, Steinberg SF (2009). p66Shc links alpha1-adrenergic receptors to a reactive oxygen species-dependent AKT-FOXO3A phosphorylation pathway in cardiomyocytes. Circ Res.

